# Evaluation of virtual patient cases for teaching diagnostic and management skills in internal medicine: a mixed methods study

**DOI:** 10.1186/s13104-018-3463-x

**Published:** 2018-06-05

**Authors:** Samira Jeimy, Jenny Yujing Wang, Lisa Richardson

**Affiliations:** 1 Division of Clinical Immunology and Allergy, Department of General Internal Medicine, Western University, B3-110, 268 Grosvenor Street, London, ON N6A 1M1 Canada; 2 Department of General Internal Medicine, University Health Network - Toronto General Hospital, University of Toronto, EN 14-218, 200 Elizabeth Street, Toronto, ON M5G 2C4 Canada

**Keywords:** Virtual patients, Medical education, CanMEDS, Medical curriculum, Internal medicine

## Abstract

**Objective:**

The virtual patient (VP) is a computer program that simulates real-life clinical scenarios and allows learners to make diagnostic and therapeutic decisions in a safe environment. Although many VP cases are available, few focus on junior trainees as their target audience. In addition, there is wide variability in trainees’ clinical rotation experiences, based on local practice and referral patterns, duty hour restrictions, and competing educational requirements. In order to standardize clinical exposure and improve trainees’ knowledge and perceived preparedness to manage core internal medicine cases, we developed a pool of VP cases to simulate common internal medicine presentations. We used quantitative and qualitative analyses to evaluate the effectiveness of one of our VP cases among medical trainees at University of Toronto. We also evaluated the role of VP cases in integrated teaching of non-medical expert competencies.

**Results:**

Despite modest effects on knowledge acquisition, a majority of participants enjoyed using VP cases as a resource to help them prepare for and reinforce clinical experiences. Cognitive interactivity and repetitive practice were particularly appreciated by study participants. Trainees perceived VP cases as a useful resource as their learning can be customized to their actions within the case, resulting in unique learning trajectories.

**Electronic supplementary material:**

The online version of this article (10.1186/s13104-018-3463-x) contains supplementary material, which is available to authorized users.

## Introduction

The rapid evolution of medical knowledge, decreased time for medical training, and ethical concerns about patients as educational subjects have increased the complexity of medical decision making and medical training [1, 2]. The virtual patient (VP) is a computer program that simulates real-life clinical scenarios and allows learners to emulate the roles of health care providers to make clinical decisions (reviewed in [3, 4]). While VP cases are available widely online, few focus on medical students and junior residents as their target audience.

In addition to the knowledge and technical expertise medical trainees must acquire, there are intrinsic competencies with significant impact on health-care delivery and patient satisfaction [5]. In the 1990s, the Royal College of Physicians and Surgeons of Canada developed the “Canadian Medical Education Directives for Specialists” (CanMEDS) framework [reviewed in 6]. Training programs have implemented curricula to integrate the framework [6, 7]; however, there remains a paucity of literature on effective means of integrating the CanMEDS framework in medical education.

To standardize clinical exposure and improve trainees’ knowledge and perceived preparedness to manage core internal medicine cases, we developed a pool of VP cases to simulate internal medicine presentations. We used quantitative and qualitative analyses to evaluate the effectiveness of one of our VP cases. We also evaluated the role of VP cases as a tool for the integrated teaching of CanMEDS competencies.

## Main text

### Methods

#### Module design

We selected a VP case from a pool developed by physicians at the University of Toronto. Each module begins with defined learning objectives, followed by the case and guided questions. Drop-down menus provide suggested responses to questions, and discussion points highlight concepts pertinent to evidence-based medicine and key psychosocial factors. For this study, we used a case on diagnosis and management of upper gastrointestinal bleed (UGIB).

#### Module evaluation

We invited University of Toronto trainees to participate in VP case evaluation. Trainees completed questionnaires about demographic items and rated their perceived confidence in diagnostic and management abilities (Additional file 1: Additional materials—Questionnaires). Items were measured on a 5-point scale ranging from 1 (“poor” confidence) to 5 (“excellent” confidence). Next, we randomized trainees to complete a VP case (intervention arm) or PowerPoint presentation (control arm). After module completion, participants again completed the confidence questionnaire, and a 10-item multiple-choice test assessing knowledge acquisition (Additional file 1: Additional materials—Questionnaires).

We invited trainees who completed the post-test to participate in audiotaped focus groups to provide open-ended feedback, especially related to their experiences in learning the integrated non-medical expert CanMEDS roles. We conducted two focus groups (5 participants each) with medical students and residents, using a semi-structured interview method. Each meeting was between 60 and 120 min in duration. Meetings were audiotaped and transcribed.

The methods were approved by the institutional research ethics board at the University of Toronto. Written consent was obtained from all participants.

#### Data analysis

We analyzed responses to the multiple-choice questions with the Wilcoxon signed-rank test using Excel software. Free text responses were analyzed using grounded theory to identify common themes. Two authors (SJ and JW) independently transcribed and analyzed the focus group audio recordings using the “framework” technique (described in [8]).

## Results

### Quantitative analysis

A total of 52 participants completed the study. Baseline characteristics were similar between groups (Table 1). A majority had used online learning modules, but most of these were non-interactive (Table 1). Participants differed in their perceived confidence in diagnosing UGIB, with lower baseline ability to diagnose and manage UGIB in trainees who completed the VP case (Table 1).

**Table 1:** Participant characteristics (% of participants in each intervention group)

	VP Case (n = 23)	PowerPoint (n = 29)	Chi square *P* value
Level of training			0.799
1–2 years medical student	13	17	
3–4 years medical student	61	52	
Resident	26	31	
Time from last IM rotation			0.965
No previous IM rotation	26	24	
> 6 months	13	10	
3–6 months	13	17	
< 3 months	26	21	
Currently in IM rotation	22	28	
Initial objectives			N/A
Review knowledge	87	90	
Acquire new medical expert knowledge	65	52	
Improve non-medical expert competency	17	10	
Application of knowledge	52	66	
Recruited for research	4	0	
Previous exposure to virtual patients			0.313
None	87	76	
Any virtual patient cases	13	24	
Self evaluation of ability to:			
Diagnose UGIB			0.064
Excellent	0	3	
Very good	43	28	
Satisfactory	35	59	
Unremarkable	4	10	
Poor	17	0	
Manage UGIB			0.394
Excellent	0	3	
Very good	22	14	
Satisfactory	48	66	
Unremarkable	9	10	
Poor	22	7	
Handover			0.340
Excellent	4	3	
Very good	26	14	
Satisfactory	48	69	
Unremarkable	13	14	
Poor	9	0	
Write admission orders			0.284
Excellent	4	3	
Good	26	10	
Satisfactory	43	55	
Unremarkable	13	28	
Poor	13	3	
OGD consent			0.518
Excellent	0	0	
Very good	13	10	
Satisfactory	22	41	
Unremarkable	43	31	
Poor	22	17	

*VP* virtual patient, *UGIB* upper gastrointestinal bleed, *OGD* oesophago-gastro-duodenoscopy

Table 2 compares average objective knowledge acquisition and clinical reasoning scores from participants’ post-module tests. Trainees performed similarly in each test question, including those pertaining to non-medical-expert CanMEDS competencies (Table 2). Change in level of confidence for clinical hand-over trended towards being higher in the VP-case arm (Mann–Whitney P = 0.051, Table 2). Overall, there were no significant differences in participants’ perceived confidence (Additional file 2: Tables S1 and Additional file 3: Table S2).

**Table 2:** Comparing objective assessment scores (median [IQR1, IQR3])

Question topics	VP case (n = 23)	PowerPoint (n = 29)	Mann–Whitney U test P-value
Sign over	1.00 (1.00–1.00)	1.00 (1.00–1.00)	0.979
Hypovolemia signs	1.00 (0.00–1.00)	0.00 (0.00–1.00)	0.284
Most important step in management	0.00 (0.00–0.50)	0.00 (0.00–0.00)	0.650
Risk scores	1.00 (1.00–1.00)	1.00 (1.00–1.00)	0.832
Presentation of UGIB	1.00 (0.80–1.00)	0.80 (0.60–1.00)	0.274
Complications of OGD	0.75 (0.75–1.00)	0.75 (0.50–1.00)	0.210
Bad exam manoeuvre	1.00 (0.00–1.00)	1.00 (0.00–1.00)	0.335
High risk OGD lesion	0.75 (0.50–1.00)	0.75 (0.50–1.00)	0.909
Post OGD monitoring	1.00 (0.00–1.00)	0.00 (0.00–1.00)	0.073

*VP* virtual patient, *UGIB* upper gastrointestinal bleed, *OGD* oesophago-gastro-duodenoscopy

We asked participants for feedback on the VP case. Although trainees thought there was similar learning value in the VP case and PowerPoint presentation, they preferred the VP case as a learning resource (Additional file 4: Tables S3 and Additional file 5: Table S4).

### Qualitative analysis

To further evaluate how medical students and residents used the VP cases in their learning, especially pertaining to integration of non-medical-expert CanMEDS competencies, we organized two trainee focus groups. The baseline characteristics of these participants are shown in Additional file 6: Table S5. Seven categories of investigation were highlighted regarding VP cases as a learning resource and for integration of CanMEDS competencies. We divided each category into subcategories; each comment was allocated to one of these subcategories. We repeated charting for focus groups using the same subcategories. The interview technique was iterative after the first focus group. We (JW and SJ) compared individual analysis of the focus group comments, and found our analyses in agreement. The categories and subcategories of the framework technique are presented in Table 3 and below.

**Table 3:** Focus group categories

Categories	Subcategories
Residents want practical resources beyond traditional curriculum	Want concise, evidence-based, clinically relevant information
	Place to practice skills without consequences
Medical students at different levels have different learning needs	Preclinical students are focused on tips/skills
	Preclinical students want to practice experience of real world before clerkship
	Clerks are focused on knowledge/medical expert content
	Clerks want to practice application of knowledge
	Difficult to meet needs with any one type of learning resource
Appreciated elements of IMCE cases	High quality, comprehensive
	Realistic
	Practical delivery of clinically relevant details
	Provides an approach
	Evidence-based
	Interactive
	Optional curriculum resource
Suggestions for improvement	Cases are too long, with too many details e.g. scoring systems
	Link to multimedia (videos, images, Apps)
	Include extra information like scoring systems as optional links
	Increase interactivity
General challenges in the current use of CanMEDS in medical education	The way CanMEDS breaks down the concept of the physician is reductionist, not organic
	Portfolio—allows debriefing on challenging cases, but rigid format
	CanMEDS is useful for educators to plan curriculum but may be inherently challenging to teach
VP cases and CanMEDS	VP cases may be a useful resource to integrate CanMEDS roles
Simulations cannot replace real world experience of patient care	Some skills are still better learned via practice and experience

#### Category 1. Trainees are looking for practical resources beyond didactic lectures

Focus group participants emphasized limited time to learn a vast amount of knowledge. The transition points from pre-clerkship to clerkship, and from senior medical student to intern, was felt to be especially challenging.

*I think one of the hardest parts when you are beginning is … we get inundated with so much information. We don’t know what is important and what is not.*

#### Category 2. Learning needs differ based on level of training

Pre-clinical students were looking to gain experience with a practical approach to a clinical presentation.

*I’m focusing less about the UGIB content and more about the experience – I think I took a lot out of it that way*

Most junior medical students wanted repeated practice with ward skills. Senior medical students confirmed that they felt inadequately prepared, which detracted from their learning experience.

*Things like handover, writing admission orders, writing a prescription, etc. - we didn’t get taught that until the week before clerkship started.*

Senior medical students were looking for efficient ways to refresh their knowledge, especially in preparation for their licensing exams. They emphasized that an interactive platform would be more effective than passive review of lectures or of the scientific literature.

The consensus was that learning resources which provide concise, practical, evidence-based information would be useful to help build confidence in diagnostic and management skills. Trainees emphasized the importance of a simulation setting where they can safely practice skills without consequences on patient care.

*It is nice to go through early in clerkship, to have a place to safely practice things without judgement or killing a patient.*

#### Category 3. VP cases are a useful adjunct to didactic lectures

In comparison to commonly-used resources, medical students appreciated that the VP case simulated a real world clinical scenario.

*Toronto Notes is … comprehensive but a book of lists with no emphasis on what’s common, what you should prioritize. This realistic case scenario which takes you through the steps in practical terms is more useful.*

Residents appreciated the teaching of practical, clinically-relevant details.

*I like the specifics - like doses and timelines, like 72 hours, how many milligram. At a resident level that’s what we need to know.*

Participants also liked the integration of evidence-based medicine, and appreciated the user-friendly, interactive aspect of the case.

*And the format you used with the dropdown menus – they gave you a chance to think about the question and then the answer was there.*

#### Category 4. Suggestions for improvement

Trainees felt the case was lengthy and contained extraneous detail. Participants also wanted a greater level of interactivity.

#### Category 5. There are challenges to current approaches to CanMEDS training

Participants felt that the way CanMEDS breaks down the concept of the physician is reductionist, and not realistic.

*CanMEDS is trying to make an abstract thing concrete and it does not make sense. If you try to focus on communicator role in our job as a physician, it is not doing it justice. We communicate all the time, it is hard to take it out of context and isolate it.*

#### Category 6. VPs may represent a useful tool for integrating CanMEDS

The consensus was that VP cases may be a useful resource to integrate CanMEDS roles in medical education. Trainees appreciated that they did not realize they were learning CanMEDS competencies throughout the VP case.

*It was a surprise. For example, writing admission orders. Those are really useful for clerkship. Nice way to integrate it without being explicit.*

They also liked that multiple competencies were covered with one concept.

*I like that about [the] cases. Like handover includes…communication, collaboration…*

#### Category 7. Simulations cannot replace real world experience of patient care

Most trainees felt that, although VP cases provided a useful adjunct, many of the CanMEDS competencies are best achieved through real world experience.

## Discussion

Our goal in creating VP cases was to facilitate transition between the role of a senior medical student and a first year internal medicine resident. This was based on consensus among colleagues and studies reporting that 41–60% of medical graduates feel clinically unprepared after medical school graduation [9–11]. Interactive VP cases allows medical educators to facilitate learning in an environment that does not compromise patient safety [12]. We hoped that our VP cases could complement the medical curricula to help trainees become comfortable with assessing and managing common, key presentations in a protected environment.

Our results indicated that VP cases did not significantly affect knowledge acquisition, for both medical expert and non-medical expert CanMEDS topics (Table 2). This is consistent with a meta-analysis of 201 studies summarizing the effect of internet-based instruction for medical trainees [13]. Despite its modest effects on knowledge acquisition, a majority of participants enjoyed using VP cases as a resource to help them prepare for, and reinforce clinical experiences. Trainees’ preference for using VP cases, especially over traditional curriculum adjuncts, is important, as learner engagement can significantly improve effectiveness of technology-enhanced simulation [14]. Other features of simulation-based training shown to be effective in medical education, including cognitive interactivity and repetitive practice [13], were aspects of our VP case appreciated by study participants. It is possible that trainees perceive VP cases as a useful resource as their learning can be customized to their actions within the case, resulting in unique learning trajectories. For example, in our study junior trainees focused on learning an approach to the consultation process, whereas senior trainees reviewed their medical knowledge. In addition, junior trainees concentrated on non-medical expert CanMEDS competencies, such as writing admission orders, whereas senior trainees enjoyed learning about evidence-based medicine. Based on current adult learning theories, such personalized and interactive instruction methods may be more powerful and efficient than didactic education [15].

Although trainees agreed that non-medical expert CanMEDS roles are important, they consistently expressed dissatisfaction with existing CanMEDS curricula, finding the approaches reductionist and artificial. Trainees appreciated incorporation of CanMEDS topics in the VP case, and especially that multiple CanMEDS competencies were introduced without disrupting the flow of the case. VP cases may provide an exciting new arena where CanMEDS competencies can be introduced or reinforced.

There are several advantages to integrating VP cases in medical education, including cost benefits, cases that closely match real-life situations, the ability to create collections of similar cases, seamless integration of CanMEDS competency training, and the ability to create VP cases with which trainees should ideally gain competence. Future work will concentrate case enhancements based on feedback, and cases that can provide real-time feedback or introduce different challenges based on training levels. We hope to create a larger pool of cases to allow for standardization in trainees’ exposure to common and atypical internal medicine presentations.

## Limitations

Our analysis is limited by a small sample size and selective participation. Another limitation is the use of self-assessment to evaluate changes in knowledge and confidence in managing UGIB, although analysis of objective knowledge scores corroborated subjective reports. Lastly, our study was limited to trainees at University of Toronto, and used one VP case to extrapolate data. It would be interesting to evaluate whether our data is reproducible for different VP cases, and at other medical training programs.

## Additional files

**Additional file 1: Additional Questionaries.** Assessment of participant characteristics, self-evaluation, knowledge assessment, and case-based feedback. Questionnaires developed for the study, to assess participant demographics, perceived confidence in diagnostic and management abilities, knowledge, and feedback on the virtual patient case.

**Additional file 2: Table S1.** Change in Level of Confidence after Intervention (Median Change in Likert Scale Rating [IQR1, IQR3]). Trainees’ perceived self-confidence in diagnostic and management abilities, measured on a 5-point rating scale ranging from 1 (“poor” confidence) to 5 (“excellent” confidence). Median change before and after completing the virtual patient case are reported.

**Additional file 3: Table S2.** Participant Characteristics for Participants who Completed Pre-test Only vs. Pre- and Post- Test (% of Participants in Each Intervention Group). Demographic characteristics and perceived confidence of participants who completed the pre-test only, with those who completed both the pre- and post- tests, to confirm that these groups were not significantly different.

**Additional file 4: Table S3.** Participant Case Evaluation (% of Participants in Each Intervention Group). Trainees’ evaluation of the virtual patient case, based on questionnaires developed for the study.

**Additional file 5: Table S4.** Themes in Free-Text Feedback. Trainees’ free-text evaluation of the virtual patient case.

**Additional file 6: Table S5.** Focus group participant characteristics. Demographic features of trainees who participated in focus groups.

## Abbreviations

VP: virtual patient
CanMEDS: Canadian Medical Education Directives for Specialists
UGIB: upper gastrointestinal bleed
