# Supplementary material for: Evaluation of virtual patient cases for teaching diagnostic and management skills in internal medicine: a mixed methods study
Source: BMC Res Notes. 2018 Jun 5;11:357. doi: 10.1186/s13104-018-3463-x (PMC5989465; doi:10.1186/s13104-018-3463-x)
Supplement: Supplementary file 1 — Additional file 1: Additional Questionaries. Assessment of participant characteristics, self-evaluation, knowledge assessment, and case-based feedback. Questionnaires developed for the study, to assess participant demographics, perceived confidence in diagnostic and management abilities, knowledge, and feedback on the virtual patient case. [file 13104_2018_3463_MOESM1_ESM.doc]

**Questionnaires. Assessment of participant characteristics, self-evaluation, knowledge assessment, and case-based feedback.**

**1) Baseline Questionnaire**

***A. Participant Characteristics***

1. What is your level of training?
   1. 1-2yr Medical Student
   2. 3^rd^ Year Medical Student
   3. 4^th^ Year Medical Student
   4. 1^st^ Year Internal Medicine Resident
   5. 1^st^ Year Non-Internal Medicine Resident
   6. Other: _____________
2. When was your most recent Internal Medicine rotation?
   1. Have not yet started your first Internal Medicine rotation
   2. Currently in your first internal medicine rotation
   3. <3 months ago
   4. 3 – 6 months ago
   5. > 6 months ago
3. What were your initial objectives for completing these cases? (Please CHECK ALL options that apply)
   - Review your knowledge of the topics covered by these cases
   - Learn more about the Medical Expert topics covered by these cases
   - Learn more about the NON-Medical Expert topics covered by these cases
   - Learn to apply knowledge to a clinical scenario
   - Other: _____________
4. What other electronic learning modules have you used before? (Please CHECK ALL options that apply)
   - None
   - Non-interactive learning modules e.g. videos, PowerPoint presentations
   - Other IMCE cases
   - Other interactive virtual patient case series, including: _____________­­­_______

***B. Self-Evaluation Questions***

1. How would you rate your ability to diagnose UGIB?
   1. Excellent
   2. Very good
   3. Satisfactory
   4. Unremarkable
   5. Poor
2. How would you rate your ability to manage a patient with UGIB?
   1. Excellent
   2. Very good
   3. Satisfactory
   4. Unremarkable
   5. Poor
3. How would you rate your ability to effectively hand over patient information?
   1. Excellent
   2. Very good
   3. Satisfactory
   4. Unremarkable
   5. Poor
4. How would you rate your ability to write admission orders?
   1. Excellent
   2. Very good
   3. Satisfactory
   4. Unremarkable
   5. Poor
5. How would you rate your ability to consent patients for an esophagogastroduodenoscopy?
   1. Excellent
   2. Very good
   3. Satisfactory
   4. Unremarkable
   5. Poor

**2) Final Questionnaire**

***A. Self-Evaluation Questions***

1. How would you rate your ability to diagnose UGIB?
2. Excellent
3. Very good
4. Satisfactory
5. Unremarkable
6. Poor
7. How would you rate your ability to manage a patient with UGIB?
8. Excellent
9. Very good
10. Satisfactory
11. Unremarkable
12. Poor
13. How would you rate your ability to effectively hand over patient information?
14. Excellent
15. Very good
16. Satisfactory
17. Unremarkable
18. Poor
19. How would you rate your ability to write admission orders?
20. Excellent
21. Very good
22. Satisfactory
23. Unremarkable
24. Poor
25. How would you rate your ability to consent patients for an esophagogastroduodenoscopy?
26. Excellent
27. Very good
28. Satisfactory
29. Unremarkable
30. Poor

***B. Objective Assessment Questions***

Q. Which of the follow best describes the information you should include about a patient when verbally signing over patient information?

i. stable or not, code status

ii. identifying information

iii. general course in hospital

iv. new events of the day

v. upcoming possibilities and management plan

vi. all the tests done for the patient

a. i, ii, iii

b. i, ii, iii, iv

c. i, ii, iii, iv, v

d. i, ii, iii, iv, v, vi

e. everything you know about the patient

Answer: c – stable or not, code status, identifying information, general course in hospital, new events of the day, overall health status now, upcoming possibilities and management plan, tasks to complete overnight, opportunity for questions for the individual you are signing over to

Q. Which of the following are the two most useful signs for hypovolemia due to blood loss?

i. orthostatic hypotension

ii. orthostatic tachycardia

iii. severe orthostatic dizziness

iv. mild orthostatic dizziness

v. dry axilla

a. i and ii

b. i and iii

c. ii and iii

d. ii and iv

e. iii and v

Answer: c – According to the JAMA article on assess for hypovolemia the most useful findings for hypovolemia due to blood loss are **severe** orthostatic dizziness (i.e. dizziness that prevents the measurement of up-right vitals signs) or orthostatic tachycardia (>=30 bpm increase in HR from supine to standing). SN for having either finding is only 22% for moderate blood loss (450 to 630mL) and is 97% for large blood loss (630 to 1150mL), with a SP of 98%.

Q. Of the following, which is the most important step in managing a stable patient with non-variceal upper GI bleed?

a. give IV proton-pump inhibitor

b. give oral proton-pump inhibitor

c. esophagogastroduodenoscopy

d. give IV octreotide

e. nasogastric lavage

Answer: c. esophagogastroduodenoscopy – PPI is often started BEFORE EGD but current evidence has not shown a difference in outcomes when it is given in this setting. Conversely, PPI is effective in preventing rebleeding and reducing mortality when given AFTER EGD. There is no significant difference between oral and IV forms. IV octreotide is used in the setting of variceal bleeding. Nasogastric lavage may be considered to help clear the stomach of blood but EGD is still the most important for definitive diagnosis and treatment.

Q. Which of the follow are scoring systems that are specifically used to risk stratification patients with peptic ulcer disease?

i. TIMI Score

ii. Blatchford Score

iii. Standford Score

iv. Rockall Score

v. ABCD_2_ Score

a. ii, iii, iv

b. i, v

c. ii, iv

d. i, ii, iii

e. i, ii, iv

Answer: c. ii and iv

Q. Upper GI bleed can present with:

i. hematemesis

ii. melena

iii. hematochezia

iv. shortness of breath

v. incidental lab findings

a. i and ii only

b. i, ii, iii

c. ii, iii

d. i, ii, iii, iv

e. i, ii, iii, iv, v

Answer: e. all of the above

Q. Which of the following are serious complications of esophagogastroduodenoscopy that should be discussed with patients?

i. perforation

ii. bleeding

iii. sedation-related cardiopulmonary events

iv. contrast allergies

v. aspiration

a. i, ii, iv, v

b. i, ii, iii, v

c. i, ii, iii, iv

d. i, ii, iv

e. i, ii, iii, iv, v

Answer: b. No contrast is used so there is no risk of contrast allergy. However, there can be risk of allergy

to e.g. anaesthetics

Q. In assessing patients with suspected upper GI bleeds, which of the following physical exam maneuvers is least recommended (i.e. weakest evidence)?

a. assessing vitals

b. fecal occult blood test

c. digital rectal exam

d. nasogastric aspirate

e. abdominal exam

Answer: b. fecal occult blood test – this test have very low sensitivity (so not helpful in ruling out disease) and since you suspect an overt bleed already, you are going to conduct further investigation e.g. EGD and so FOBT will not change your management

Q. At discharge, follow-up plan for a patient treated acutely for NSAID-induced peptic ulcer disease should include:

a. pantoprazole 40 mg PO BID x 1 month and then daily

b. follow-up with family physician

c. outpatient Helicobacter pylori testing and treatment if positive

d. a and b

e. all of the above

Answer: e. all of the above

Q. Which of the following are considered high-risk endoscopic lesions according to the Forrest Classification?

i. spurt blood

ii. ooze blood

iii. non-bleeding visible vessel

iv. adherent clot

v. flat, pigmented spot

a. i

b. i, ii

c. i, ii, iii

d. i, ii, iii, iv

e. i, ii, iii, iv, v

Answer: d. i, ii, iii, iv

Q. Mrs. Y is a 57yro female with a history of osteoarthritis and chronic NSAID use, presenting with upper GI bleed. Endoscopy showed a 1 cm gastric ulcer associated with a non-bleeding visible vessel. After endoscopy, she should be:

a. discharged home with oral proton-pump inhibitors.

b. admitted to the GIM ward for observation for 24 hours.

c. admitted to the GIM ward for observation for 72 hours.

d. admitted to a monitored setting e.g. step-up for at least 1 day and then can be transferred to the ward for 2 additional days of monitoring.

e. admitted to a monitored setting e.g. step-up for at least 72 hours and then consider step down to the ward for 2 additional days of monitoring.

Answer: d – admitted to a monitored setting e.g. step-up for at least 1 day and then can be transferred to the ward for 2 additional days of monitoring. This is the current suggested management guideline for a high-risk endoscopic lesion.

***C. Case-Based Feedback***

Please select the MOST APPROPRIATE option, unless otherwise instructed.

1. I found this case user friendly.
2. Strongly agree
3. Somewhat agree
4. Neutral
5. Somewhat disagree
6. Strongly disagree
7. While working on this case, I felt I had to make the same decisions a doctor would make in real life.
8. Strongly agree
9. Somewhat agree
10. Neutral
11. Somewhat disagree
12. Strongly disagree
13. While working on this case, I felt I were the doctor caring for this patient.
14. Strongly agree
15. Somewhat agree
16. Neutral
17. Somewhat disagree
18. Strongly disagree
    - 1. I felt that the case was at the appropriate level of difficulty for my level of training.
19. Strongly agree
20. Somewhat agree
21. Neutral
22. Somewhat disagree
23. Strongly disagree
    - 1. After completing this case, I feel better prepared to confirm a diagnosis and exclude differential diagnoses in a real life patient with this complaint.
24. Strongly agree
25. Somewhat agree
26. Neutral
27. Somewhat disagree
28. Strongly disagree
    - 1. After completing this case I feel better prepared to care for a real life patient with this complaint.
29. Strongly agree
30. Somewhat agree
31. Neutral
32. Somewhat disagree
33. Strongly disagree
    - 1. Overall, working through this case was a worthwhile learning experience.
34. Strongly agree
35. Somewhat agree
36. Neutral
37. Somewhat disagree
38. Strongly disagree
    - 1. How long did it take for you to go through this case?
    1. <15 minutes
    2. 15-30 minutes
    3. 30 – 45 minutes
    4. 45 – 60 minutes
    5. more than 60 minutes
       1. How did you find the length of this case?
39. Much too short
40. A little too short
41. Just right
42. A little too long
43. Much too long

10. I appreciated the inclusion of clerkship tips (e.g. writing admission orders, how to consent) in the case

1. Strongly agree
2. Somewhat agree
3. Neutral
4. Somewhat disagree
5. Strongly disagree

11. I recognized that these tips related to non-medical expert CanMEDS roles.

1. Strongly agree
2. Somewhat agree
3. Neutral
4. Somewhat disagree
5. Strongly disagree

12. I prefer learning CanMEDS roles in the context of a clinical case as opposed to in separate modules.

1. Strongly agree
2. Somewhat agree
3. Neutral
4. Somewhat disagree
5. Strongly disagree

13. I found the case overall to be:

1. Excellent
2. Better than expected
3. Meets expectations
4. Unremarkable
5. Poor

14. Specific strengths of the case:

15. Specific weaknesses of the case:

16. Any additional comments:
