# Supplementary material for: Evaluation of virtual patient cases for teaching diagnostic and management skills in internal medicine: a mixed methods study
Source: BMC Res Notes. 2018 Jun 5;11:357. doi: 10.1186/s13104-018-3463-x (PMC5989465; doi:10.1186/s13104-018-3463-x)
Supplement: Supplementary file 2 — Additional file 2: Table S1. Change in Level of Confidence after Intervention (Median Change in Likert Scale Rating [IQR1, IQR3]). Trainees’ perceived self-confidence in diagnostic and management abilities, measured on a 5-point rating scale ranging from 1 (“poor” confidence) to 5 (“excellent” confidence). Median change before and after completing the virtual patient case are reported. [file 13104_2018_3463_MOESM2_ESM.docx]

**Additional file 2: Table S1: Change in Level of Confidence after Intervention (Median Change in Likert Scale Rating [IQR1, IQR3])**

|  | **VP Case (n = 23)** | **PowerPoint (n = 29)** | **Mann-Whitney U Test P-value** |
| --- | --- | --- | --- |
| Diagnose UGIB | 1.0 (0.00-1.50) | 1.0 (0.0-1.0) | 0.108 |
| Manage UGIB | 1.0 (0.5-1.0) | 1.0 (0.0-1.0) | 0.302 |
| Handover | 1.0 (0.0-1.0) | 0.0 (0.0-1.0) | 0.051 |
| Write Admission Orders | 1.0 (0.0-1.0) | 1.0 (0.0-1.0) | 0.457 |
| OGD Consent | 1.0 (1.0-2.0) | 1.0 (0.0-2.0) | 0.526 |
