# Supplementary material for: Evaluation of virtual patient cases for teaching diagnostic and management skills in internal medicine: a mixed methods study
Source: BMC Res Notes. 2018 Jun 5;11:357. doi: 10.1186/s13104-018-3463-x (PMC5989465; doi:10.1186/s13104-018-3463-x)
Supplement: Supplementary file 3 — Additional file 3: Table S2. Participant Characteristics for Participants who Completed Pre-test Only vs. Pre- and Post- Test (% of Participants in Each Intervention Group). Demographic characteristics and perceived confidence of participants who completed the pre-test only, with those who completed both the pre- and post- tests, to confirm that these groups were not significantly different. [file 13104_2018_3463_MOESM3_ESM.docx]

**Additional file 3: Table S2: Participant Characteristics for Participants who Completed Pre-test Only vs. Pre- and Post- Test (% of Participants in Each Intervention Group)**

|  | **Pre-test Only (n = 170)** | **Pre- and Post- Test (n = 52)** | **Chi Square P-value** |
| --- | --- | --- | --- |
| **Level of Training** |  |  | 0.174 |
| 1-2yr Medical Student | 27 | 15 |  |
| 3-4yr Medical Student | 54 | 56 |  |
| Resident | 20 | 29 |  |
| **Time From Last IM Rotation** |  |  | 0.387 |
| No Previous IM Rotation | 39 | 25 |  |
| > 6 Months | 9 | 12 |  |
| 3-6 Months | 12 | 15 |  |
| < 3 Months | 15 | 23 |  |
| Currently in IM Rotation | 25 | 25 |  |
| **Initial Objectives** |  |  | N/A |
| Review Knowledge | 82 | 89 |  |
| Acquire New Medical Expert Knowledge | 53 | 58 |  |
| Improve Non-Medical Expert Competency | 19 | 14 |  |
| Application of Knowledge | 51 | 60 |  |
| Recruited for Research | 1 | 2 |  |
| **Previous Exposure to Virtual Patients** |  |  | 0.644 |
| None | 84 | 81 |  |
| Any Virtual Patient Cases | 17 | 19 |  |
| ***Self Evaluation of Ability To:*** |  |  |  |
| **Diagnose UGIB** |  |  | 0.170 |
| Excellent | 3 | 8 |  |
| Very Good | 28 | 8 |  |
| Satisfactory | 37 | 48 |  |
| Unremarkable | 21 | 35 |  |
| Poor | 12 | 2 |  |
| **Manage UGIB** |  |  | 0.179 |
| Excellent | 2 | 2 |  |
| Very Good | 18 | 17 |  |
| Satisfactory | 40 | 58 |  |
| Unremarkable | 21 | 10 |  |
| Poor | 18 | 14 |  |
| **Handover** |  |  | 0.300 |
| Excellent | 4 | 4 |  |
| Very Good | 22 | 14 |  |
| Satisfactory | 45 | 60 |  |
| Unremarkable | 19 | 19 |  |
| Poor | 11 | 4 |  |
| **Write Admission Orders** |  |  | 0.136 |
| Excellent | 2 | 4 |  |
| Very Good | 21 | 17 |  |
| Satisfactory | 35 | 50 |  |
| Unremarkable | 23 | 21 |  |
| Poor | 20 | 8 |  |
| **OGD Consent** |  |  | 0.496 |
| Excellent | 0 | 0 |  |
| Very Good | 14 | 12 |  |
| Satisfactory | 27 | 33 |  |
| Unremarkable | 31 | 37 |  |
| Poor | 29 | 19 |  |
