# Supplementary material for: Evaluation of virtual patient cases for teaching diagnostic and management skills in internal medicine: a mixed methods study
Source: BMC Res Notes. 2018 Jun 5;11:357. doi: 10.1186/s13104-018-3463-x (PMC5989465; doi:10.1186/s13104-018-3463-x)
Supplement: Supplementary file 4 — Additional file 4: Table S3. Participant Case Evaluation (% of Participants in Each Intervention Group). Trainees’ evaluation of the virtual patient case, based on questionnaires developed for the study. [file 13104_2018_3463_MOESM4_ESM.docx]

**Additional file 4: Table S3: Participant Case Evaluation (% of Participants in Each Intervention Group)**

|  | **VP Case (n = 23)** | **PowerPoint (n = 29)** | **Chi Square P-value** |
| --- | --- | --- | --- |
| **User Friendly** |  |  | 0.044 |
| Strongly Agree | 65 | 21 |  |
| Somewhat agree | 35 | 45 |  |
| Neutral | 0 | 31 |  |
| Somewhat disagree | 0 | 3 |  |
| Strongly disagree | 0 | 0 |  |
| **Realism: Decision Making** |  |  | 0.140 |
| Strongly agree | 52 | 14 |  |
| Somewhat agree | 43 | 48 |  |
| Neutral | 4 | 24 |  |
| Somewhat disagree | 0 | 10 |  |
| Strongly disagree | 0 | 3 |  |
| **Realism: Patient Care** |  |  | 0.292 |
| Strongly agree | 35 | 7 |  |
| Somewhat agree | 43 | 41 |  |
| Neutral | 17 | 31 |  |
| Somewhat disagree | 4 | 7 |  |
| Strongly disagree | 0 | 14 |  |
| **Level Appropriate** |  |  | 0.288 |
| Strongly agree | 61 | 24 |  |
| Somewhat agree | 26 | 41 |  |
| Neutral | 9 | 24 |  |
| Somewhat disagree | 0 | 10 |  |
| Strongly disagree | 4 | 0 |  |
| **Confidence: Diagnosis** |  |  | 0.184 |
| Strongly agree | 43 | 17 |  |
| Somewhat agree | 52 | 55 |  |
| Neutral | 4 | 28 |  |
| Somewhat disagree | 0 | 0 |  |
| Strongly disagree | 0 | 0 |  |
| **Confidence: Management** |  |  | 0.112 |
| Strongly agree | 57 | 14 |  |
| Somewhat agree | 43 | 59 |  |
| Neutral | 0 | 24 |  |
| Somewhat disagree | 0 | 3 |  |
| Strongly disagree | 0 | 0 |  |
| **Learning Value** |  |  | 0.552 |
| Strongly agree | 65 | 31 |  |
| Somewhat agree | 30 | 48 |  |
| Neutral | 4 | 17 |  |
| Somewhat disagree | 0 | 3 |  |
| Strongly disagree | 0 | 0 |  |
| **Time Required** |  |  | 0.073 |
| <15 minutes | 9 | 34 |  |
| 15-30 minutes | 52 | 28 |  |
| 30 – 45 minutes | 22 | 24 |  |
| 45 – 60 minutes | 9 | 14 |  |
| more than 60 minutes | 9 | 0 |  |
| **Evaluation of Case Length** |  |  | 0.202 |
| Much too short | 0 | 0 |  |
| A little too short | 0 | 14 |  |
| Just right | 74 | 69 |  |
| A little too long | 22 | 17 |  |
| Much too long | 4 | 0 |  |
| **Like Clerkship Tips** |  |  | 0.356 |
| Strongly agree | 78 | 59 |  |
| Somewhat agree | 17 | 21 |  |
| Neutral | 0 | 17 |  |
| Somewhat disagree | 4 | 3 |  |
| Strongly disagree | 0 | 0 |  |
| **Recognize Non-medical Expert CanMEDS Roles** |  |  | 0.857 |
| Strongly agree | 52 | 17 |  |
| Somewhat agree | 22 | 48 |  |
| Neutral | 22 | 21 |  |
| Somewhat disagree | 4 | 14 |  |
| Strongly disagree | 0 | 0 |  |
| **Prefer Integrated CanMEDS Teaching** |  |  | 0.936 |
| Strongly agree | 57 | 55 |  |
| Somewhat agree | 22 | 28 |  |
| Neutral | 22 | 14 |  |
| Somewhat disagree | 0 | 0 |  |
| Strongly disagree | 0 | 3 |  |
| **Overall Evaluation** |  |  | 0.072 |
| Excellent | 48 | 17 |  |
| Better than expected | 39 | 48 |  |
| Meets expectations | 13 | 28 |  |
| Unremarkable | 0 | 7 |  |
| Poor | 0 | 0 |  |
