# Supplementary material for: Evaluation of virtual patient cases for teaching diagnostic and management skills in internal medicine: a mixed methods study
Source: BMC Res Notes. 2018 Jun 5;11:357. doi: 10.1186/s13104-018-3463-x (PMC5989465; doi:10.1186/s13104-018-3463-x)
Supplement: Supplementary file 5 — Additional file 5: Table S4. Themes in Free-Text Feedback. Trainees’ free-text evaluation of the virtual patient case. [file 13104_2018_3463_MOESM5_ESM.docx]

**Additional file 5: Table S4: Themes in Free-Text Feedback**

|  | **VP Case (n = 23)** | **PowerPoint (n = 29)** |
| --- | --- | --- |
| **Strengths** | - **Realistic (22%):** “Believable scenario that would very likely come up in real life in the near future (while on Medicine).” - **Detailed/Comprehensive (17%):** “Included information I had not learned about before – e.g. the two scoring systems for PUD as well as what makes a lesion high or low risk”, “Wow - very thorough and comprehensive” - **Organized/Good Format (17%):** “The step by step format was very user friendly and easy to follow.” - **Socratic Style** **(13%):** “The fact that the objectives were presented as questions, and only once you clicked could you see the answers - this enabled you to really think about what the answer would be.” - **Relevance/Helpful** **(9%):** “Clinical relevance. I know when I did GIM, had a lot of cases of UGIB and this would definitely have been helpful.” - **Interactive (9%):** “Interactive” - **Application (4%):** “Given general info, then given specific case info and asked to apply it. - **Clear/Concise** **(4%):** “Clear, concise” - **Subtle CanMEDS Integration (4%):** “I did not notice the CanMEDS integration.” - **EBM (4%):** “Good integration of EBM.” | - **Comprehensive (28%):** “PowerPoint presentation was comprehensive” - **Relevant (17%):** “Common presentation” - **Learning Tips (10%):** “Tips for writing admission orders and patient handover was extremely useful (I'll be referring to these notes in other clerkship rotations).” - **Concise (21%):** “Clear, concise summary of relevant clinical information related to GI bleed.” - **Good/well written (14%):** “Very well written case - high quality” - **Teaches decision making (3%):** “Understand decision making process” - **Visual format (3%):** “Format of the presentation: layout, visuals, etc” - **Applicable to diff stages (3%):** “Applicable to variety of stages of training” - **Can save (3%):** “I can save the ppt” - **Practical (3%):** “Contained a great deal of practical information (without too much pathophysiology) which is relevant for a clinical setting.” |
| **Weaknesses** | - **None (35%):** “None” - **Too Long (26%):** “A little too long” - **Too Detailed (13%):** “A bit too long/detailed” - **Should Indicate Relative Importance (4%):** “Lack of indication of relative importance of different pieces of information made it overwhelming.” - **Broken link (4%):** “The links didn't work (e.g. to the abbreviations or other blue highlighted text)” | - **Excessive Detail (17%):** PowerPoint presentation was dense - **Not Realistic (17%):** “PowerPoint presentation had minimal patient cases”, “Very didactic” - **Not Engaging (14%):** “Format not as engaging (reading through PowerPoint slides)”, “I didn't enjoy this at all.”, “The ppt slides are easy to flip through "tune out" and not read carefully.” - **Long (10%):** “It's very long” - **No Application of Knowledge (7%):** “Not enough opportunity to make patient-related decisions.” - **Lack Visual Aids (3%):** “No pictures” - **Formatting Problems (3%):** “Some of the tables/footnotes were overlapping on my PC” - **None (3%):** “No weakness identified” |
