# Supplementary material for: Evaluation of virtual patient cases for teaching diagnostic and management skills in internal medicine: a mixed methods study
Source: BMC Res Notes. 2018 Jun 5;11:357. doi: 10.1186/s13104-018-3463-x (PMC5989465; doi:10.1186/s13104-018-3463-x)
Supplement: Supplementary file 6 — Additional file 6: Table S5. Focus group participant characteristics. Demographic features of trainees who participated in focus groups. [file 13104_2018_3463_MOESM6_ESM.docx]

**Additional file 6: Table S5: Focus group participant characteristics**

| **Focus group** | **Type of participant** | **Specialty** | **Gender** | **Year of medical school graduation** | **Level of appointment** | **Number of four-week internal medicine rotations completed** |
| --- | --- | --- | --- | --- | --- | --- |
| 1 | Resident | Internal Medicine | F | 2008 | PGY2 | 8 |
| 1 | Resident | Internal Medicine | F | 2008 | PGY1 | 5 |
| 1 | Resident | Internal Medicine | M | 2008 | PGY2 | 8 |
| 1 | Resident | Internal Medicine | M | 2008 | PGY2 | 8 |
| 1 | Resident | Pathology | M | 2008 | PGY2 | 2 |
| 1 | Resident | Internal medicine | F | 2008 | PGY2 | 8 |
| 2 | Medical student | Undifferentiated | F | 2017 | Yr 1 medical school | 0 |
| 2 | Medical student | Undifferentiated | M | 2017 | Yr 1 medical school | 0 |
| 2 | Medical student | Undifferentiated | M | 2015 | Yr 3 medical school | 2 |
| 2 | Medical student | Matched to Internal Medicine | M | 2014 | Yr 4 medical school | 3 |
| 2 | Medical student | Matched to Family Medicine | M | 2014 | Yr 4 medical school | 2 |
